# The Dietetic Value of Beer and Stout

**Published:** 1909-05-01

**Authors:** 


### The Hospital

A Journal of

The Medical Sciences and Hospital Administration.

New Series. No. 113, Vol. V. [No. 1185, Vol. XLVI.] SATURDAY, MAY 1, 1909.

### THE DIETETIC VALUE OF BEER AND STOUT.

The report of the Special Commission on Beer which we published last week has caused much interest in medical circles and outside them. It is the result of many months of close investigation and impartial inquiry. For many years, to an increasing extent, beer has been subject to an unjust prejudice due to an impression that its consumption is harmful rather than beneficial to many people. It is time that beer should be justified in public estimation by proving, as our Commission does, its great nutritive value, apart altogether from its alcohol content. The fact is that good, properly made beer is a beverage containing a very small amount of alcohol and a relatively large amount of nutritive material. It is time that the erroneous view that beer has no nutritive value in itself, and merely consists of a beverage upon which a certain portion of the community intoxicates itself, should be exposed and discredited. The results of our Commission show that beer is *par excellence* the nutritive alcoholic beverage. All beverages because they contain alcohol should not be regarded in the same light. The spirit nipper is committing quite a different act from the beer drinker; in fact, beer is much further removed, from the point of view of its alcohol content, from some wines and all spirits than it is from ginger-beer.

When a man drinks good beer he drinks and eats at the same time, just as when he eats a bowl of soup. The terms "eat" and "drink" are curiously but inconsistently used as connoting the difference between what is merely quenching our thirst and what is actually consuming nourishment. Our Commissioners point out a man might more properly be said to eat beer than to eat certain kinds of soup, or indeed water-melon. Their report will enable members of the medical profession and the public to understand clearly what constitutes good beer, and where and how they may obtain it. Beer drinkers, the numbers of whom we hope will increase considerably as the result of the researches of our Commissioners, are now in a position to protect themselves from bad beers, and we hold the view that it would be infinitely better for the well-being of the people of these islands as a whole if they were to select beer as their habitual drink, rather than wines or spirits. Climacteric conditions have a good deal to do with the dietetic value of substances used for allaying thirst. Our Commissioners properly drive home that, when a man drinks beer or stout habitually, he is not only drinking but eating, a fact which has not been sufficiently recognised in recent years. These beverages contain all the elements of a typical diet, with the exception of fat, and in a proportion approximately physiological. Our Commissioners remind us that if the worth of a food is measured by its calorimetric value the fact is that a glass of good ale is approximately as nourishing as a glass of milk, and that a quart of good beer is nearly equivalent to a quarter of a pound of beef.

The fashion in regard to beers and stout is changing and there has been a very considerable growth in the consumption of the latter in the last few years, owing to its increasing use for nutritive purposes in cases of influenza and other ailments where the system requires building up. Beer and stout are of course not suitable foods for the obese, or for the subjects of renal disorders or glycosuria or diabetes; but where there is no counter-indication the habitual use of beer or stout in moderate quantities is better calculated to benefit residents in these islands than the habitual use of either wines or spirits. Another important medicinal aspect of beer and stout, and especially of the latter, is their hypnotic action. Stout is one of the most harmless and best hypnotics we possess, and is often far more efficacious in the treatment of insomnia than drugs. Part of their action is due, our Commissioners remind us, to the hypnotic principle contained in hops, and the more heavily these beverages are hopped the more marked is their hypnotic action. An average dose for the purpose is half a pint, and this should be taken the last thing at night, as long after an early dinner or supper as possible. Another point in favour of beer and stout as beverages is that the likelihood of their containing pathogenic organisms is too extremely remote. This gives them, like tea and coffee, a great advantage over milk, for they are all prepared under conditions which render pollution by infective bacteria extremely improbable.

These remarks must not be misconstrued. We are quite alive, as our Commissioners are, to the tremendous harm done by the abuse of alcohol and the good done by sympathising with temperance. At the same time, with the philanthropist's desire to improve mankind, we have the scientist's regard for fact. Upon untruth no enduring fabric, however philanthropic its motive, can ever be securely built. The fact is that good, properly made beer is a beverage containing a very small amount of alcohol and a relatively large amount of nutritive material. It is too often forgotten that beverages like ginger-beer and koumiss contain slightly more than 1 per cent. of alcohol, whilst there are beers on the market containing only 2 or 3 per cent. of this drug. Our Commissioners give some interesting comparisons between beer, tea, and beef-tea. Those comparisons indicate that beer compares favourably with both these products, and our Commissioners properly insist that it would be difficult to find a meal at once simpler and more nutritive than a crust of bread and cheese, or bread and butter, or both, and beer.

